# Prevalence of the most common pathogenic variants in three genes for inborn errors of metabolism associated with sudden unexpected death in infancy: a population-based study in south Brazil

**DOI:** 10.1590/1678-4685-GMB-2019-0298

**Published:** 2020-07-24

**Authors:** Dévora N. Randon, Fernanda Sperb-Ludwig, Fernanda S. L. Vianna, Ana P. P. Becker, Carmen R. Vargas, Angela Sitta, Alexia N. Sant’Ana, Ida V. D. Schwartz, Fernanda H. de Bitencourt

**Affiliations:** 1 Universidade Federal do Rio Grande do Sul (UFRGS), Programa de Pós-Graduação em Genética e Biologia Molecular, Porto Alegre, RS, Brazil.; 2 Hospital de Clínicas de Porto Alegre (HCPA), Centro de Pesquisa Experimental, Basic Research and Advanced Investigations in Neurosciences (BRAIN), Porto Alegre, RS, Brazil.; 3 Hospital de Clínicas de Porto Alegre (HCPA), Centro de Pesquisa Experimental, Laboratório de Medicina Genômica, Porto Alegre, RS, Brazil.; 4 Universidade Federal do Rio Grande do Sul (UFRGS), Faculdade de Medicina, Porto Alegre, RS, Brazil.; 5 Universidade Federal do Rio Grande do Sul (UFRGS), Faculdade de Farmácia, Porto Alegre, RS, Brazil.; 6 Hospital de Clínicas de Porto Alegre (HCPA), Serviço de Genética Médica, Porto Alegre, RS, Brazil.; 7 Universidade Federal do Rio Grande do Sul (UFRGS), Instituto de Biociências, Porto Alegre, RS, Brazil.; 8 Universidade Federal do Rio Grande do Sul (UFRGS), Departamento de Genética, Porto Alegre, RS, Brazil.; 9 Hospital de Clínicas de Porto Alegre (HCPA), Instituto Nacional de Genética Médica Populacional (INAGEMP), Porto Alegre, RS, Brazil.

**Keywords:** Citrullinemia type I, long-chain 3-hydroxyacyl-CoA dehydrogenase deficiency, mut0 methylmalonic acidemia, pathogenic variants, prevalence

## Abstract

Citrullinemia type 1 (CTLNI), long-chain 3-hydroxyacyl-CoA dehydrogenase deficiency (LCHADD), and mut^0^ methylmalonic acidemia (mut^0^ MMA) are inborn errors of metabolism (IEMs) associated with sudden unexpected death in infancy (SUDI). Its most common pathogenic variants are: c.1168G&gt;A (CTLNI, *ASS1* gene), c.1528G&gt;C (LCHADD, *HADHA* gene), c.655A&gt;T and c.1106G&gt;A (mut^0^ MMA, *MUT* gene). Considering the absence of estimates regarding the incidence of these diseases in Brazil, this study sought to investigate the prevalence of its main pathogenic variants in a healthy population in the southern region of the country. A total of 1,000 healthy subjects from Rio Grande do Sul were included. Genotyping was performed by real-time PCR. Individuals found to be heterozygous for c.1528G&gt;C underwent further acylcarnitine profile analysis by tandem mass spectrophotometry. Allele and genotype frequencies were calculated considering Hardy-Weinberg equilibrium. The c.1528G&gt;C variant was detected in heterozygosity in two subjects (carrier frequency = 1:500; allele frequency = 0.001; minimum prevalence of LCHADD = 1: 1,000,000), whose acylcarnitine profiles were normal. Variants c.1168G&gt;A, c.655A&gt;T, and c.1106G&gt;A were not identified. These results denote the rarity of these IEMs in Southern Brazil, highlighting the need to expand the investigation of IEMs in relation to infant morbidity and mortality within the country.

Sudden unexpected death in infancy (SUDI) refers to the death of a child that occurs suddenly and unexpectedly during the first year of life, and represents one of the leading causes of post-neonatal death (Krous *et al.*, 2004). Inborn errors of metabolism (IEMs) are estimated to account for 0.9% to 6% of such events (Boles *et al.*, 1998; Chace *et al.*, 2001; van Rijt *et al.*, 2016). Among the IEMs known to be associated with SUDI, citrullinemia type 1 (CTLNI; MIM #215700), long-chain 3-hydroxyacyl-CoA dehydrogenase deficiency (LCHADD; MIM #609016) and mut^0^ methylmalonic acidemia (mut^0^ MMA; #MIM 25100), among other autosomal recessive IEMs, are treatable and identifiable by neonatal screening (van Rijt *et al.*, 2016).

CTLNI is a urea cycle disorder caused by deficiency of the enzyme argininosuccinate synthetase (ASS, EC 6.3.4.5) due to pathogenic variants in the *ASS1* gene (Beaudet *et al.*, 1986). Among these, c.1168G&gt;A (p.Gly390Arg) in exon 15 accounts for up to 62% of alleles in European patients diagnosed with the disorder (Diez-Fernandez *et al.*, 2017). LCHADD, a disorder of fatty-acid beta-oxidation, is caused by pathogenic variants in the *HADHA* gene, which encodes the alpha subunit of the mitochondrial trifunctional protein (MTP; EC 1.1.1.211) (IJlst *et al.*, 1994). Variant c.1528G&gt;C (p.Glu510Gln) in exon 15 was observed in 87–91% of European patients with clinically overt disease (Nedoszytko *et al.*, 2017). Certain variants in the *MUT* gene, which codes for the enzyme methylmalonyl-CoA mutase (MUT; EC 5.4.99.2), are responsible for mut^0^ MMA, a disorder of propionate catabolism (Ledley *et al.*, 1988). The most frequent implicated variants are c.655A&gt;T (p.Asn219Tyr), in exon 3, and c.1106G&gt;A (p.Arg369His), in exon 6, representing approximately 25% (Acquaviva *et al.*, 2005) and 10% (Forny *et al.*, 2016) of alleles in European patients, respectively.

Considering that the aforementioned IEMs are not included in the Brazilian nationwide neonatal screening program and that there are no estimates regarding their incidence in the country, we aimed at investigating the presence of the most prevalent pathogenic variants associated with these conditions in a healthy population in the state of Rio Grande do Sul, southern Brazil.

This was an observational, cross-sectional study with a convenience sampling strategy.

The sample size was estimated at 980 individuals. Calculation was performed in WINPEPI (PEPI-for-Windows, version 11.65), based on the known proportion of heterozygotes for variant c.1528G&gt;C (*HADHA*) in European populations (den Boer *et al.*, 2000; Joost *et al.*, 2012), an error rate of 0.3%, statistical power of 80%, alpha = 0.05, and 95% confidence interval (95%CI).

Participants were recruited from the blood bank at Hospital de Clínicas de Porto Alegre, Rio Grande do Sul, Brazil, a tertiary teaching hospital affiliated with Universidade Federal do Rio Grande do Sul, between August 2017 and March 2019. Each participant completed a form designed to collect data on date and place of birth and ancestry (European or non-European).

From each participant, a 5 mL sample of peripheral blood was collected into an EDTA-containing tube and stored at -20 °C. Genomic DNA was extracted with the commercially available Easy-DNA Kit (Invitrogen, Carlsbad, CA, USA), following the manufacturer-provided protocol.

Genomic DNA was analyzed by real-time polymerase chain reaction (RT-PCR) with TaqMan genotyping (Thermo Fisher) in StepOne and QuantStudio 3 systems (Thermo Fisher), performed in accordance with the manufacturer’s instructions. Custom TaqMan genotyping assays were ordered from Applied Biosystems (Foster City, CA, USA), based on the reference sequences NG_011542.1 (*ASS1*), NG_007121.1 (*HADHA*), and NG_007100.1 (*MUT*).

Individuals heterozygous for the c.1528G&gt;C variant (*HADHA*) also underwent acylcarnitine profile analysis by tandem mass spectrometry (MS/MS) using peripheral blood on filter paper samples, based on the protocol established by Rashed *et al.* (1997).

Statistical analyses were performed in SPSS Version 22.0 (IBM Corp., Armonk, NY, USA). The carrier frequency was obtained by calculating the ratio of heterozygous individuals to the total number of individuals analyzed, and reported with a 95%CI. Gene and genotype frequencies were calculated assuming Hardy-Weinberg equilibrium in the population.

The study protocol was approved by the local Research Ethics Committee, in accordance with Brazilian regulations for human subject research (under reference number 17-0249) and all participants provided written informed consent. Individuals who were found to be heterozygous were convoked for genetic counseling purposes.

The study sample consisted of 1,000 voluntary blood donors, with a mean age of 36.6 ± 12.1 years (range 19–69 years) (Table 1).

**Table 1:** Characteristics of the study sample (N=1,000).

Variable	N (%)
*Sex*	
Male	504 (50.4%)
Female	496 (49.6%)
*Birthplace*	
Rio Grande do Sul	922 (92.2%)
Porto Alegre	470 (47%)
Other municipalities	452 (45.2%)
Other states	75 (7.5%)
Other countries	3 (0.3%)
*Ancestry*	
European	639 (63.9%)
German	272 (27.2%)
Italian	202 (20.2%)
Portuguese	110 (11.0%)
Other European	55 (5.5%)
No European ancestry	252 (25.2%)
Unknown	109 (10.9%)

Variants c.1168G&gt;A (*ASS1*), c.655A&gt;T and c.1106T&gt;A (*MUT*) were not identified in the analyzed sample. Two subjects (0.2%) were found to be heterozygous for the c.1528G&gt;C (*HADHA*) variant (Table 2), which corresponds to a carrier frequency of 1:500 (95%CI 0.02%–0.72%). The resulting allele frequency for c.1528G and c.1528C were 0.9980 (95%CI 0.9949-0.9995) and 0.001 (95%CI 0.0001-0.0036), respectively. The estimated minimum prevalence for LCHADD as a consequence of the c.1528C allele in Rio Grande do Sul was 1:1,000,000. The acylcarnitine profiles of both individuals heterozygous for c.1528G&gt;C were normal.

**Table 2:** Characteristics of individuals heterozygous for variant c.1528G>C (*HADHA*).

Subject	Sex	Age (years)	Birthplace	European ancestry
1	Female	52	Porto Alegre	Yes
2	Female	29	Porto Alegre	No

The Brazilian population is one of the most ethnically heterogeneous of the world, as the result of five centuries of miscegenation among indigenous, European, and African populations. In the southern region, European ancestry predominates (Parra *et al.*, 2003; Moura *et al.*, 2015) as our data corroborate. This predominance of European ancestry might suggest that the prevalence of the variants of interest in the population of Rio Grande do Sul would be similar to that reported in European countries.

The absence of the c.1168A allele (*ASS1*) could be indicating its rarity in the region, in agreement with the information available in the Exome Aggregation Consortium (ExAC) and in the Genome Aggregation Database (gnomAD), which show a frequency of ~0.0003, both for European and global populations. However, these databases do not include information about Brazilian individuals. The Brazilian Online Archive of Mutations (ABraOM), which catalogs exon variants from 609 healthy elderly from the city of São Paulo, in Southeast Brazil, shows a similar frequency, of 0.0016 (Naslavsky *et al.*, 2017).

The prevalence of c.1168A was previously described for USA and Argentine populations (Table 3). In the USA, Bardos *et al.* (2019) determined a carrier frequency of 1:383 and a prevalence of 1:575,000, contradicting the estimated overall incidence of CTLNI (~1:250,000) (Summar *et al.*, 2013). The authors have suggested that this discrepancy could be explained by a possible overestimation of CTLNI prevalence reported in the literature, or by an overestimate of the frequency of the pathogenic variants included in their assay (Bardos *et al.*, 2019). In comparison, in the city of Villa Mercedes, Argentina, Laróvere *et al.* (2012) reported a carrier frequency of 1:25 and a prevalence of 1:2,427. Additionally, the authors determined a CTLNI occurrence of 57% for the offspring of couples consisting of two heterozygous individuals, a much higher frequency than expected for autosomal recessive disorders, which suggests a preferential transmission of the c.1168A allele. This phenomenon was previously proposed by Kleijer *et al.* (2006), based on the high rate of affected fetuses (39.5%), diagnosed from a total of 91 pregnancies at 1 in 4 risk. They proposed that the preferential transmission of any citrullinemic allele might arise from a protective role of ASS deficiency in haploid mutant sperm cells against the possibly detrimental or apoptotic effect of nitric oxide, produced normally from arginine by nitric oxide sintetase. The possibility of a preferential transmission highlights the relevance of this pathogenic variant in relation to early diagnosis and genetic counseling for populations at risk.

**Table 3:** Characteristics of the study sample (N=1,000).

Variant	Population	Number of heterozygotes/Number of analyzed individuals	Carrier frequency	References
c.1168G>A	Argentina	7/172	1:25	Laróvere *et al*., 2012
	United States	29/11132	1:383	Bardos *et al*., 2019
	Brazil (São Paulo)	2/609	1:305	Naslavsky *et al*., 2017
	Brazil (Rio Grande do Sul)	0/1000	0	This study
c.1528G>C	China	0/1200	0	Zhu *et al*., 2005
	Estonia	6/1040	1:173	Joost *et al*., 2012
	Finland	5/1200	1:240	Tyni and Pihko, 1999
	Finland	9/1637	1:181	Pastinen *et al*., 2001
	Finland (North)	1/365	1:365	Pastinen *et a*l., 2001
	Finland (South)	3/492	1:164	Pastinen *et al.*, 2001
	Finland (East)	2/385	1:193	Pastinen *et al*., 2001
	Finland (West)	3/392	1:132	Pastinen *et al.*, 2001
	Netherlands	3/2047	1:680	den Boer *et* al., 2000
	Poland (children)	22/4137	1:189	Piekutowska-Abramczuk *et al*., 2010
	Poland (adults)	36/5877	1:163	Nedoszytko *et* al., 2017
	Poland (Pomerania, children)	41/2976	1:73	Piekutowska-Abramczuk *et* al., 2010
	Poland (Pomerania, adults)	4/413	1:103	Nedoszytko *et al*., 2017
	Poland (Kashubia, adults)	18/1023	1:57	Nedoszytko *et* al., 2017
	Brazil (São Paulo)	2/609	1:305	Naslavsky *et al*., 2017
	Brazil (Rio Grande do Sul)	2/1000	1:500	This study

The allele frequency estimated in this study for the variant c.1528G&gt;C (*HADHA*) is in agreement with ExAC and gnomAD data, which show a frequency of 0.0012 for the global population and 0.0016 for Europe (non-Finnish). Moreover, in São Paulo, the frequency is also 0.0016 (Naslavsky *et al.*, 2017). The prevalence of this allele has been described in several populations (Table 3). We found a carrier frequency of 1:500, which exceeds the estimated frequency for the Netherlands (1:680) (den Boer *et al.*, 2000), but is lower than reported for other European populations. The highest frequency was observed in northern Poland, with a probable founder effect in the population of Kashubia (Piekutowska-Abramczuk *et al.*, 2010; Nedoszytko *et al.*, 2017). In comparison, the allele was absent in all 1,200 individuals analyzed in a study in Beijing (Zhu *et al.*, 2005). Based on the frequency of c.1528G&gt;C, the birth prevalence of LCHADD is predicted to be 1:118,336 in Poland (Piekutowska-Abramczuk *et al.*, 2010) and 1:91,700 in Estonia (Joost *et al.*, 2012). Our findings suggest that the minimum prevalence of LCHADD in Rio Grande do Sul would be 1:1,000,000. According to data from neonatal MS/MS screening programs, the prevalence of this condition ranges from 1:1,148,000 in Korea to 1:840,000 in Japan (Shibata *et al.*, 2018). It should be noted that MS/MS cannot distinguish between general mitochondrial trifunctional protein deficiency (MIM #609015) and LCHADD.

The c.655A&gt;T variant (*MUT*) presents an allele frequency of ~0.00005 in the global population, according to ExAC and gnomAD data, being found only in non-Finnish European populations and Ashkenazi Jews. Likewise, the overall frequency of c.1106G&gt;A (*MUT*) is reported as ~0.00004 in ExAC and ~0.00006 in gnomAD. In non-Finnish European populations, it is ~0.00004 in ExAC and ~0.00007 in gnomAD. Both variants are absent from the ABraOM database (Naslavsky *et al.*, 2017). According to USA neonatal screening data, the prevalence of mut^0^ MMA is ~1:100,000 (Feuchtbaum *et al.*, 2012). To the best of our knowledge, there are as yet no other estimates for the frequency of these variants in healthy populations.

This was the first study to evaluate the prevalence of the most frequent pathogenic variants in the genes implicated in CTLNI, LCHADD, and mut^0^ MMA—three inborn errors of metabolism associated with SUDI—in a Brazilian healthy population. The low estimated prevalence of the variants is thought to denote the rarity of these disorders in Rio Grande do Sul. However, one must take into account the limitations arising from the size of the analyzed cohort, since it does not allow for the detection of low frequency variants. Another fact to consider is related to the allele heterogeneity associated with *ASS1*, *HADHA*, and *MUT* genes. Despite the preponderance of European ancestry in southern Brazil, other pathogenic variants, which cannot be identified through the genotyping method used in the present study, may occur with a frequency higher than that established for other geographical areas currently covered in the literature. The findings of this study are particularly relevant in the context of early diagnosis and genetic counseling, and underscore the need to expand IEMs investigation in relation to infant morbidity and mortality within the territory.
